# Cloning a Chloroplast Genome in *Saccharomyces cerevisiae* and *Escherichia coli*

**DOI:** 10.21769/BioProtoc.5162

**Published:** 2025-01-20

**Authors:** Emma Jane Lougheed Walker, Bogumil Jacek Karas

**Affiliations:** Biochemistry Department, Western University, London, Canada

**Keywords:** Synthetic biology, Chloroplast, Genome engineering, Genome replacement, Yeast assembly, Cloning, Transformation, *Phaeodactylum tricornutum*, *Escherichia coli*, PCR

## Abstract

Chloroplast genomes present an alternative strategy for large-scale engineering of photosynthetic eukaryotes. Prior to our work, the chloroplast genomes of *Chlamydomonas reinhardtii* (204 kb) and *Zea mays* (140 kb) had been cloned using bacterial and yeast artificial chromosome (BAC/YAC) libraries, respectively. These methods lack design flexibility as they are reliant upon the random capture of genomic fragments during BAC/YAC library creation; additionally, both demonstrated a low efficiency (≤ 10%) for correct assembly of the genome in yeast. With this in mind, we sought to create a highly flexible and efficient approach for assembling the 117 kb chloroplast genome of *Phaeodactylum tricornutum*, a photosynthetic marine diatom. Our original article demonstrated a PCR-based approach for cloning the *P. tricornutum* chloroplast genome that had 90%–100% efficiency when screening as few as 10 yeast colonies following assembly. In this article, we will discuss this approach in greater depth as we believe this technique could be extrapolated to other species, particularly those with a similar chloroplast genome size and architecture.

Key features

• Large fragments of the chloroplast genome can be readily amplified through PCR from total algal DNA isolate.

• Assembly protocol can be completed within a day, and yeast colonies harboring chloroplast genomes can be obtained in as few as 4–5 days.

• Cloned genomes isolated from yeast transformants can be moved to *Escherichia coli* through electroporation.

## Graphical overview


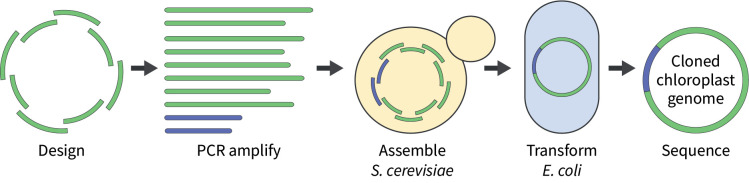


**The chloroplast genome is split into overlapping fragments (green), which are then PCR-amplified along with a suitable cloning vector (blue).** The fragments are transformed into *Saccharomyces cerevisiae* for assembly, where they will recombine to form the cloned chloroplast genome. DNA can be isolated from yeast transformants and electroporated into *Escherichia coli* for further analysis. Ultimately, to confirm the capture of the cloned genome, DNA from representative yeast and/or *E. coli* transformants is sequenced.

## Background

The ability to assemble, deliver, and install whole synthetic genomes provides the utmost control when engineering an organism. However, synthesizing large fragments of DNA (i.e., > 10 kb) is still prohibitively expensive for most academic labs, whilst cloning and transforming partial or whole chromosomes remains technically challenging, if not unexplored, in most species. The model systems for whole-genome replacement have been limited to comparatively simple organisms with well-established DNA manipulation techniques (e.g., viruses [1,2], bacteria [3,4], and *Saccharomyces cerevisiae* [5]). Given the exciting possibilities that whole-genome replacement offers, there is a growing demand to establish other biological chassis capable of large-scale engineering feats [6]. Establishing a photosynthetic chassis is of particular interest to the biotechnology and bioeconomy sectors, as photosynthetic organisms can capture and use atmospheric CO_2_ as a carbon source, forgoing the need for energetically costly carbon inputs.

Photosynthetic eukaryotes possess distinct nuclear, chloroplast, and mitochondrial genomes. The aforementioned organelles typically contain multiple copies of a single, highly reduced chromosome, making these genomes more feasible to synthesize and “replace” compared to nuclear chromosomes, which are larger and more complex. The chloroplast genome is a particularly interesting target for genome replacement as the organelle serves as a hub for several cellular biosynthesis pathways of industrial relevance (e.g., starch, amino acid, and fatty acid synthesis). Despite this, there are only two published papers demonstrating the potential for cloning an entire chloroplast genome outside of the organelle. The first paper describes the capture of the *Zea mays* chloroplast genome (140 kb) in *Saccharomyces cerevisiae* [7]. Here, high molecular weight DNA was isolated from *Z. mays*, then shorn and ligated into a yeast artificial chromosome (YAC) backbone. More than 10,000 yeast clones were screened, with only one clone demonstrating what appeared to be the complete *Z. mays* chloroplast genome. The second paper describes the assembly of the *Chlamydomonas reinhardtii* chloroplast genome (204 kb) from six overlapping fragments, of which four had been previously captured in a bacterial artificial chromosome (BAC) library [8]. Here, 3 out of 30 yeast transformants contained the correctly assembled chloroplast genome following yeast assembly. Creating BAC/YAC libraries is technically demanding, time consuming, and does not permit the utmost design flexibility for assembling large constructs as it is dependent upon whichever fragments were generated during the library preparation stage. An efficient and tractable strategy for assembling whole chloroplast genomes is a necessary first step to achieve the full potential of this unique chassis.

We sought to design and test alternative assembly strategies for cloning whole chloroplast genomes. *Phaeodactylum tricornutum* was selected as our model organism due to its ease of propagation and rapidly growing toolbox for genetic engineering. Here, we present a strategy for cloning the *P. tricornutum* chloroplast genome that demonstrated 90%–100% efficiency when screening as few as 10 yeast colonies and 3 *Escherichia coli* colonies following whole-genome assembly and transformation, respectively. We believe this efficient and tractable method for cloning the *P. tricornutum* chloroplast genome could be extrapolated to other photosynthetic eukaryotes, particularly those with similar genome sizes and architectures (e.g., *Thalassiosira pseudonana*).

## Materials and reagents

**Biological materials**

1. *P. tricornutum* liquid culture [Culture Collection of Algae, the University of Texas at Austin (UTEX), catalog number: 646]

2. *E. coli* TransforMax EPI300 cells (LGC Biosearch Technologies, Lucigen, catalog number: EC300110)

3. *S. cerevisiae* VL6-48 culture [American Type Culture Collection (ATCC), catalog number: MYA-3666]

**Reagents**

1. 1 kb DNA ladder (New England Biolabs, catalog number: N3232L)

2. 2-Mercaptoethanol (Sigma-Aldrich, catalog number: M3148)

3. Adenine hemisulfate salt (Sigma-Aldrich, catalog number: A2545)

4. Agar A (Bio Basic, catalog number: FB0010)

5. Agarose (FroggaBio, catalog number: A87-500G)

6. Bacteriological peptone (BioShop, catalog number: PEP403)

7. Bacto agar (Becton Dickinson, catalog number: 214030)

8. Bio-tryptone (BioShop, catalog number: TRP402)

9. Boric acid (Bio Basic, catalog number: BB0044)

10. Calcium chloride, dihydrate (BioShop, catalog number: CCL302)

11. Cetyltrimethylammonium bromide (CTAB) (Sigma-Aldrich, catalog number: H6269)

12. Chloramphenicol (Bio Basic, catalog number: CB0118)

13. Chloroform:isoamyl alcohol, 24:1 (Bio Basic, catalog number: CB0351)

14. Cobalt (II) sulfate, heptahydrate (Sigma-Aldrich, catalog number: CDS004010)

15. Complete media glucose broth minus tryptophan (Teknova, catalog number: C7131)

*Note: This product is no longer available; as an alternative, we suggest minimal SD base (Takara, catalog number: 630411) and -Trp DO supplement (Takara, catalog number: 630413).*

16. Complete media glucose broth minus histidine and uracil (Teknova, catalog number: C7221)

*Note: This product is no longer available; as an alternative, we suggest minimal SD base (Takara, catalog number: 630411) in addition to the minus histidine and uracil (-His/-Ura) DO supplement (Takara, catalog number: 630422).*

17. Copper (II) sulfate, pentahydrate (Bio Basic, catalog number: CDB0063)

18. Cyanocobalamin, i.e., Vitamin B12 (BioShop, catalog number: VIT271)

19. D-biotin (Bio Basic, catalog number: BB0078)

20. D-glucose (BioShop, catalog number: GLU501)

21. D-sorbitol (BioShop, catalog number: SOR508)

22. DNA gel loading dye (New England Biolabs, catalog number: B7024S)

23. *DpnI* restriction enzyme (New England Biolabs, catalog number: R0176)

24. Ethylenediaminetetraacetic acid disodium salt, dihydrate (EDTA) (Bio Basic, catalog number: EB0185)

25. Ethanol, 95% purity (Greenfield Global, catalog number: P016EA95)

26. Ethanol, anhydrous (Greenfield Global, catalog number: P006EAAN)

27. Ethidium bromide (BioShop, catalog number: ETB444)

28. Glycerol (Bio Basic, catalog number: GB0232)

29. Iron chloride, hexahydrate (Bio Basic, catalog number: FD0201)

30. Isopropanol, min. 99.5% purity (Bioshop, catalog number: SO920)

31. L-arabinose (BioShop, catalog number: ARB222)

32. Lysozyme, egg white (BioShop, catalog number: LYS702)

33. Magnesium chloride, hexahydrate (BioShop, catalog number: MAG510)

34. Manganese (II) chloride, tetrahydrate (Sigma-Aldrich, catalog number: 805930)

35. Molybdic acid, sodium salt (Bio Basic, catalog number: MB0358)

36. Nickel (II) sulfate, hexahydrate (BioShop, catalog number: NIC700)

37. Phenol:chloroform:isoamyl alcohol, 25:24:1 (Fisher Scientific, catalog number: 15593031)

38. Polyethylene glycol 8000 (Fisher Scientific, catalog number: BP233-1)

39. Potassium acetate (Bio Basic, catalog number: PRB0438)

40. Potassium bromide (BioShop, catalog number: POB333)

41. Potassium chloride (Bio Basic, catalog number: PB0440)

42. Potassium chromate (Thermo Scientific Chemicals, catalog number: AC447201000)

43. Proteinase K solution (BioShop, catalog number: PRK222)

44. RNase A (QIAGEN, catalog number: 19101)

45. Selenious acid (Thermo Scientific Chemicals, catalog number: 211176)

46. Sodium acetate (Bio Basic, catalog number: SB1611)

47. Sodium bicarbonate (Bio Basic, catalog number: SB0482)

48. Sodium chloride (BioShop, catalog number: SOD004)

49. Sodium dodecyl sulfate (BioShop, catalog number: SDS001)

50. Sodium hydroxide (BioShop, catalog number: SHY700)

51. Sodium fluoride (BioShop, catalog number: SFL001)

52. Sodium nitrate (Bio Basic, catalog number: SD0484)

53. Sodium orthovanadate (BioShop, catalog number: SOV850)

54. Sodium phosphate, dibasic (BioShop, catalog number: SPD307)

55. Sodium phosphate, monobasic (BioShop, catalog number: SPM400)

56. Sodium sulfate (BioShop, catalog number: SOS513)

57. Thiamine hydrochloride, i.e., Vitamin B1 (Sigma-Aldrich, catalog number: T4625)

58. Tris hydrochloride (Bio Basic, catalog number: TB0103)

59. Yeast extract (BioShop, catalog number: YEX555)

60. Zinc sulfate, heptahydrate (Bio Basic, catalog number: ZB2906)

61. Zymolyase, 20,000 units/g (BioShop, catalog number: ZYM001)

**Solutions**

1. Biotin stock solution, 0.1% (w/v)

2. Boric acid stock solution, 0.1% (w/v)

3. Chilled isopropanol, 100% (v/v), -20 °C

4. Chilled ethanol, 70% (v/v), -20 °C

5. Cobalt (II) sulfate stock solution, 1% (w/v)

6. Copper (II) sulfate stock solution, 0.98% (w/v)

7. Cyanocobalamin stock solution, 0.1% (w/v)

8. D-sorbitol, 1 M

9. Ethylenediaminetetraacetic acid solution, 0.5 M, pH 8.0 (EDTA)

10. Glucose, 1 M

11. Glycerol, 50% (v/v)

12. Magnesium chloride, 1 M

13. Manganese (II) chloride stock solution, 18% (w/v)

14. Molybdic acid stock solution, 0.63% (w/v)

15. Nickel (II) sulfate stock solution, 0.27% (w/v)

16. Potassium chloride, 250 mM

17. Potassium chromate stock solution, 0.194% (w/v)

18. Selenious acid stock solution, 0.13% (w/v)

19. Sodium acetate, 3 M, pH 5.2

20. Sodium fluoride stock solution, 0.1% (w/v)

21. Sodium hydroxide solution, 1 M

22. Sodium orthovanadate stock solution, 0.184% (w/v)

23. Synthetic media lacking -Trp

24. Synthetic media lacking -His/Ura

25. Tris hydrochloride, 1 M, pH 8.0 (Tris-HCl)

26. Zinc sulfate stock solution, 2.2% (w/v)

**DNA extraction**

1. Buffer P1 (see Recipes)

2. Buffer P2 (see Recipes)

3. Buffer P3 (see Recipes)

4. CTAB lysis buffer (see Recipes)

**Culturing *E. coli***

1. LB broth (see Recipes)

2. SOB broth (see Recipes)

3. SOC broth (see Recipes)

**Culturing yeast**

1. 2× YPAD broth (see Recipes)

2. Bacto-agar, 2% (w/v) (see Recipes)

3. Complete media (CM) glucose broth minus histidine and uracil (see Recipes)

**Yeast assembly**

1. SPEM solution (see Recipes)

2. Zymolase solution (see Recipes)

3. STC solution (see Recipes)

4. PEG-8000 solution (see Recipes)

5. SOS media (see Recipes)

**Culturing *P. tricornutum***

1. NP stock, 500× (see Recipes)

2. L1 trace metals stock, 1,000× (see Recipes)

3. F/2 vitamin stock solution, 2,000× (see Recipes)

4. Anhydrous salts solution, 2× (see Recipes)

5. Hydrous salts solution, 2× (see Recipes)

6. L1 media (see Recipes)

**Recipes**

**A. DNA extraction**

**1. Buffer P1**

Store at 4 °C for < 12 months. Recipe made publicly available by QIAGEN.

**Table:** 

Reagent	Final concentration	Amount
Tris-HCl (1 M, pH 8.0)	5.0 × 10^-2^ M	5 mL
EDTA (0.5 M, pH 8.0)	1.0 × 10^-2^ M	2 mL
RNAse A (100 mg/mL)	100 μg/mL	100 μL
ddH_2_O	n/a	92.9 mL
Total	n/a	100 mL

**2. Buffer P2**

Store at room temperature for < 24 months. Recipe made publicly available by QIAGEN.

**Table:** 

Reagent	Final concentration	Amount
Sodium hydroxide	2.0 × 10^-1^ M	4 g
Sodium dodecyl sulfate	1% (w/v)	5 g
ddH_2_O	n/a	Top up to 500 mL
Total	n/a	500 mL

**3. Buffer P3**

Store at room temperature for < 24 months. Recipe made publicly available by QIAGEN.

**Table:** 

Reagent	Final concentration	Amount
Potassium acetate	3 M	147.2 g
ddH_2_O	n/a	Top up to 500 mL
Total	n/a	500 mL

**4. CTAB lysis buffer**

Store at 4 °C for < 12 months. Recipe was obtained from Giguere et al. [9].

**Table:** 

Reagent	Final concentration	Amount
Sodium chloride	1.4 M	4.1 g
Tris-HCl (1 M, pH 8.0)	2.0 × 10^-1^ M	10 mL
EDTA (0.5 M, pH 8.0)	5.0 × 10^-2^ M	5 mL
CTAB	2% (w/v)	1 g
RNAse A (100 mg/mL)	250 μg/mL	125 μL
ddH_2_O	n/a	Top up to 50 mL
Total	n/a	50 mL

**B. Culturing *E. coli***

**1. LB broth**

Sterilize by autoclaving, then store at room temperature for < 12 months. To make solid media, add 1.5 g of agar A for every 100 mL of LB (1.5%, w/v) prior to autoclaving.

**Table:** 

Reagent	Final concentration	Amount
Bio-tryptone	n/a	10 g
Sodium chloride	1.71 × 10^-1^ M	10 g
Yeast extract	n/a	5 g
dH_2_O	n/a	Top up to 1,000 mL
Total	n/a	1,000 mL

**2. SOB broth**

Mix all the components, excluding the magnesium chloride, then adjust the pH to 7.0 using NaOH and/or HCl as necessary. Sterilize by autoclaving and allow to cool to room temperature. Then, aseptically add 10 mL of sterile magnesium chloride solution. The magnesium chloride solution can be sterilized by autoclaving as well. SOB broth can be stored at room temperature for < 12 months.

**Table:** 

Reagent	Final concentration	Amount
Bio-tryptone	n/a	20 g
Yeast extract	n/a	5 g
Sodium chloride	1.0 × 10^-2^ M	0.5 g
Potassium chloride, 250 mM	2.5 × 10^-3^ M	10 mL
dH_2_O	n/a	Top up to 990 mL*
Magnesium chloride, 1 M	1.0 × 10^-3^ M	10 mL
Total	n/a	1,000 mL

**3. SOC broth**

Aseptically add 20% (w/v) filter-sterilized glucose to the sterile SOB broth. We recommend preparing 50 mL sterile aliquots of this media as it is very susceptible to contamination.

**Table:** 

Reagent	Final concentration	Amount
SOB broth	n/a	1,000 mL
Glucose, 1 M	2.0 × 10^-2^ M	20 mL
Total	n/a	1,020 mL

**C. Culturing yeast**

**1. 2× YPAD broth**

Filter sterilize into a sterile glass bottle, then store at room temperature for < 12 months. D-glucose (i.e., dextrose) can burn if autoclaved, which will impact the growth of yeast. To make solid media, combine 2× YPAD with melted 2% (w/v) bacto-agar. Ensure the YPAD and bacto-agar have been equilibrated to 60 °C before combining in a 1:1 ratio to make 1% bacto-agar 1× YPAD plates. If using pre-mixed YPD agar (e.g., Sigma-Aldrich, catalog number: Y1500), combine 130 g/L of powder with dH_2_O and autoclave for 15 min at 121 °C to sterilize.

**Table:** 

Reagent	Final concentration	Amount
Yeast extract	n/a	20 g
Bacteriological peptone	n/a	40 g
D-glucose	2.2 × 10^-1^ M	40 g
Adenine hemisulfate	n/a	160 mg
dH_2_O	n/a	Top up to 1,000 mL
Total	n/a	1,000 mL

**2. Bacto-agar, 2% (w/v)**

Autoclave to sterilize, ensuring that the glass bottle is no more than 80% full to avoid boiling over. Store at room temperature for < 24 months. The recipe below lists the volume we would prepare in a 500 mL glass bottle.

**Table:** 

Reagent	Final concentration	Amount
Bacto-agar	2% (w/v)	8 g
dH_2_O	n/a	Top up to 400 mL
Total	n/a	400 mL

**3. Complete media (CM) glucose broth minus histidine and uracil**

Adjust pH to 6.0, then autoclave to sterilize; store at room temperature for < 12 months. Only include D-sorbitol if plating spheroplasted yeast (i.e., during yeast assembly); omit when plating yeast with intact cell walls. To make solid media, add 2 g of Bacto-agar for every 100 mL of media (2%, w/v). Recipe is based on the manufacturer’s guidelines (Teknova) and Karas et al. [10].

**Table:** 

Reagent	Final concentration	Amount
CM glucose broth minus histidine and uracil	n/a	28.4 g
D-sorbitol	1.0 M	182 g
Adenine hemisulfate	8.69 × 10^-4^ M	160 mg
dH_2_O	n/a	Top up to 1,000 mL
Total	n/a	1,000 mL

**D. Yeast assembly**

**1. SPEM solution**

Filter sterilize into a sterile glass bottle, then store at room temperature for < 12 months. Recipe was obtained from Karas et al. [10].

**Table:** 

Reagent	Final concentration	Amount
D-sorbitol	1.0 M	182 g
EDTA (0.5 M, pH 8.0)	1.0 × 10^-2^ M	20 mL
Sodium phosphate, dibasic	1.47 × 10^-2^ M	2.08 g
Sodium phosphate, monobasic	2.32 × 10^-3^ M	0.32 g
dH_2_O	n/a	Top up to 1,000 mL
Total	n/a	1,000 mL

**2. Zymolyase solution**

Sterilize using a syringe filter, then store in sterile 1.5 mL tubes at -20 °C for < 12 months. The efficiency of zymolyase noticeably drops with every freeze-thaw event, so we suggest storing aliquots of 45–90 μL, which is enough for 1–2 assembly reactions. Recipe was obtained from Karas et al. [10].

**Table:** 

Reagent	Final concentration	Amount
Zymolyase	400 units/mL	200 mg
Tris-HCl (1 M, pH 8.0)	1.0 × 10^-1^ M	1 mL
Glycerol (50% v/v)	25% (v/v)	10 mL
ddH_2_O	n/a	9 mL
Total	n/a	20 mL

**3. STC solution**

Filter sterilize into a sterile glass bottle, then store at room temperature for < 12 months. It is optimal to store this solution as 15 mL aliquots in sterile conical tubes. Recipe was obtained from Karas et al. [10].

**Table:** 

Reagent	Final concentration	Amount
D-sorbitol	1.0 M	182 g
Tris-HCl (1 M, pH 8.0)	1.0 × 10^-2^ M	10 mL
Calcium chloride, dihydrate	1.0 × 10^-2^ M	1.47 g
Magnesium chloride (1 M)	2.5 × 10^-3^ M	2.5 mL
dH_2_O	n/a	Top up to 1,000 mL
Total	n/a	1,000 mL

**4. PEG-8000 solution**

Adjust pH to 8.0 with sodium hydroxide solution, then filter sterilize into a sterile glass bottle; store at 4 °C for < 12 months. PEG-8000 will depolymerize over time, thereby becoming less effective for yeast assembly. This process is exacerbated if the solution is left at room temperature. It is important to check the pH of this solution ahead of its use in assembly; when stored correctly, the pH should remain at or near 8.0 for up to a year. If the pH drops below this, dispose of the solution and remake it. Recipe was obtained from Karas et al. [10].

**Table:** 

Reagent	Final concentration	Amount
PEG-8000	20% (w/v)	20 g
Calcium chloride, dihydrate	1.0 × 10^-2^ M	0.147 g
Magnesium chloride (1 M)	2.5 × 10^-3^ M	250 μL
Tris-HCl (1 M, pH 8.0)	1.0 × 10^-2^ M	1 mL
dH_2_O	n/a	Top up to 100 mL
Total	n/a	100 mL

**5. SOS media**

Filter sterilize into a sterile glass bottle, then store at room temperature for < 12 months. It is optimal to store this solution as 15 mL aliquots in sterile conical tubes. Recipe was obtained from Karas et al. [10].

**Table:** 

Reagent	Final concentration	Amount
D-sorbitol	1.0 M	182 g
Bacteriological peptone	n/a	5 g
Yeast extract	n/a	2.5 g
Calcium chloride, dihydrate	6.04 × 10^-3^ M	0.888 g
dH_2_O	n/a	Top up to 1,000 mL
Total	n/a	1,000 mL

**E. Culturing *P. tricornutum***

**1. NP stock, 500×**

Filter sterilize into a sterile glass bottle, then store at room temperature for < 24 months. Recipe was obtained from Karas et al. [11].

**Table:** 

Reagent	Final concentration	Amount
Sodium nitrate	4.4 M	37.5 g
Sodium phosphate, monobasic	1.80 × 10^-1^ M	2.5 g
ddH_2_O	n/a	Top up to 100 mL
Total	n/a	100 mL

**2. L1 trace metals stock, 1,000×**

Filter sterilize into a sterile glass bottle, then store at 4 °C for < 24 months. Recipe was obtained from Karas et al. [11].

**Table:** 

Reagent	Final concentration	Amount
Iron chloride, hexahydrate	1.17 × 10^-2^ M	3.15 g
EDTA disodium salt, dihydrate	1.17 × 10^-2^ M	4.36 g
Copper (II) sulfate stock solution, 0.98% (w/v)	9.81 × 10^-6^ M	0.25 mL
Molybdic acid stock solution, 0.63% (w/v)	7.81 × 10^-5^ M	3.0 mL
Zinc sulfate stock solution, 2.2% (w/v)	7.65 × 10^-5^ M	1.0 mL
Cobalt (II) sulfate stock solution 1% (w/v)	3.56 × 10^-5^ M	1.0 mL
Manganese (II) chloride stock solution, 18% (w/v)	9.10 × 10^-4 ^M	1.0 mL
Selenious acid stock solution, 0.13% (w/v)	1.01 × 10^-5^ M	1.0 mL
Nickel (II) sulfate stock solution, 0.27% (w/v)	1.03 × 10^-5^ M	1.0 mL
Sodium orthovanadate stock solution, 0.184% (w/v)	1.00 × 10^-5^ M	1.0 mL
Potassium chromate stock solution, 0.194% (w/v)	9.99 × 10^-6^ M	1.0 mL
ddH_2_O	n/a	Top up to 1,000 mL
Total	n/a	1,000 mL

**3. F/2 vitamin stock solution, 2,000×**

Filter sterilize into a sterile glass bottle, then store at 4 °C for < 24 months. Recipe was obtained from Karas et al. [11].

**Table:** 

Reagent	Final concentration	Amount
Thiamine hydrochloride	5.93 × 10^-4^ M	200 mg
Biotin stock solution, 0.1% (w/v)	4.09 × 10^-5^ M	10 mL
Cyanocobalamin stock solution, 0.1% (w/v)	7.38 × 10^-7^ M	1 mL
ddH_2_O	n/a	Top up to 1,000 mL
Total	n/a	1,000 mL

**4. Anhydrous salts solution, 2×**

We use this immediately for making L1 media. If necessary, it can be filter sterilized and stored at 4 °C for < 12 months. Recipe was obtained from Karas et al. [11].

**Table:** 

Reagent	Final concentration	Amount
Sodium chloride	8.38 × 10^-1^ M	24.5 g
Sodium sulfate	5.76 × 10^-2^ M	4.09 g
Potassium chloride	1.88 × 10^-2^ M	0.7 g
Sodium bicarbonate	4.76 × 10^-3^ M	0.2 g
Potassium bromide	1.68 × 10^-3^ M	0.1 g
Boric acid stock solution, 0.1% (w/v)	9.70 × 10^-4^ M	3 mL
Sodium fluoride stock solution, 0.1% (w/v)	1.43 × 10^-4^ M	300 μL
ddH_2_O	n/a	Top up to 500 mL
Total	n/a	500 mL

**5. Hydrous salts solution, 2×**

We use this immediately for making L1 media. If necessary, it can be filter sterilized and stored at 4 °C for < 12 months. Recipe was obtained from Karas et al. [11].

**Table:** 

Reagent	Final concentration	Amount
Magnesium chloride, hexahydrate	1.09 × 10^-1^ M	11.1 g
Calcium chloride, dihydrate	2.10 × 10^-2^ M	1.54 g
ddH_2_O	n/a	Top up to 500 mL
Total	n/a	500 mL

**6. L1 media**

Adjust pH to 8.0 with sodium hydroxide solution, then filter sterilize into a sterile glass bottle; store at 4 °C for < 12 months. Note that this media lacks supplemental silica (sodium metasilicate nonahydrate) as it is not necessary for *P. tricornutum* growth. Recipe was obtained from Karas et al. [11].

**Table:** 

Reagent	Final concentration	Amount
Anhydrous salts solution, 2×	1×	500 mL
Hydrous salts solution, 2×	1×	500 mL
NP stock solution, 500×	1×	2 mL
L1 trace metals solution, 1,000×	1×	1 mL
F/2 vitamin solution, 2,000×	1×	0.5 mL
Total	n/a	~1,000 mL

**Laboratory supplies**

1. 0.2 mL PCR 8-strip tubes (FroggaBio, catalog number: STF-A120-S)

2. 1.5 mL tubes (FroggaBio, catalog number: 1210-001)

3. 15 mL conical tubes (FroggaBio, catalog number: TB15-500)

4. 50 mL conical tubes (FroggaBio, catalog number: TB50-500)

5. Bottle-top filters (≥ 500 mL capacity) with 0.2 μm PES membrane (Thermo Scientific, catalog number: 09-741-07)

6. Disposable hemocytometer, Neubauer-improved chamber (Fisher Scientific, SKC, catalog number: 22-600-100)

7. Disposable plastic cuvettes (VWR, catalog number: 97000-586)

8. Electrocuvettes, 2 mm (Fisher Scientific, catalog number: FB102)

9. Erlenmeyer flasks of various sizes (e.g., 100 mL, 250 mL)

10. EZ-10 Spin Column Plasmid DNA Miniprep kit (Bio Basic, catalog number: BS614)

11. EZ-10 Spin Column PCR Products Purification Kit (Bio Basic, catalog number: BS664)

12. Large-construct kit (QIAGEN, catalog number: 12462)

13. Liquid nitrogen (available at the institution)

14. Multiplex PCR kit (QIAGEN, catalog number: 206143)

15. Petri dishes, 100 × 15 mm (VWR, catalog number: 25384-088)

16. Porcelain mortar and pestle (Fisher Scientific, catalog numbers: FB961A and FB961K)

17. PrimeSTAR GXL DNA Polymerase kit (Takara, catalog number: R050A)

18. Two-sided disposable polystyrene cuvettes, 1.5–3.0 mL volume (VWR, catalog number: 97000-586)

19. Various sizes of glass bottles that can be sterilized

20. Pipette tips: 2 μL, 200 μL, 1,000 μL

## Equipment

1. Biosafety cabinet (NuAire, model: LabGard ES NU-540)

2. Centrifuge with 15–50 mL tube capacity (e.g., Eppendorf, model: 5810, catalog number: 05-413-332)

3. Cryogenic dewar for liquid nitrogen

4. DeNovix spectrophotometer (DeNovix, model: DS-11, catalog number: DS-11)

5. Gel documentation system (Bio-Rad, model: ChemiDoc Imaging System, catalog number: 12003153)

6. Gel electrophoresis systems (Fisher Scientific, models: Owl EasyCast B1, B2 and B3, discontinued)

7. Gel power system (Fisher Scientific, catalog number: FB300Q)

8. Meker burner (Flinn Scientific, catalog number: AP1021)

9. Microcentrifuge with 1.5 mL tube capacity (Eppendorf, model: 5415C, discontinued)

10. pH probe (Sartorius, model: pHBasic+, discontinued)

11. Pipettes: P2, P20, P100, and P1000

12. Room or chamber at 18 °C with cool white light (i.e., blue-shifted) set to an intensity of 50–65 PPFD

13. Stationary/shaking incubators that can be set to 37 °C and 30 °C (Benchmark Scientific, catalog number: H1001-M)

14. Stir plate (Benchmark Scientific, catalog number: H4000-HS)

15. T100 thermal cycler (Bio-Rad, catalog number: 1861096)

## Software and datasets

1. Benchling; free use, web-based platform (https://www.benchling.com/)

2. Image Lab; free use, application from Bio-Rad (version 6.1.0 build 7, standard edition)

3. Primer3web; free use, web-based platform (https://primer3.ut.ee/)

4. Sequenced and annotated *P. tricornutum* chloroplast genome; GenBank accession number EF067920.1, created by Oudot-Le Secq et al. [12]

## Procedure

**A. Designing the primers for PCR amplification of the chloroplast genome**

1. Upload the sequenced and annotated chloroplast genome into Benchling or any other DNA manipulation software (e.g., SnapGene, Geneious). The *P. tricornutum* chloroplast genome can be accessed through GenBank (GenBank accession number EF067920.1; Oudot-Le Secq et al. [12]).

2. Roughly divide the genome into fragments that are between 5 and 20 kb and overlap by 400 bp.

**Figure 1.**
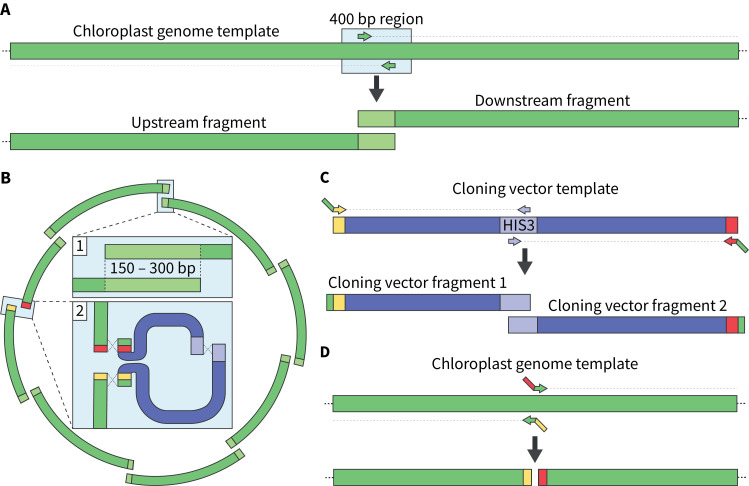
Designing primers to PCR-amplify the chloroplast genome as overlapping fragments. A) Primers are positioned within a 400 bp region of the chloroplast genome using Primer3, which generates optimized ~20 bp primers. The reverse primer will be used to amplify the upstream fragment, whereas the forward primer will be used to amplify the downstream fragment. **B)** The chloroplast genome should be split into fragments that overlap by 150–300 bp at every junction (box 1) except for where the cloning vector will integrate (box 2). Here, the chloroplast genome will be amplified using primers that will enable the integration of the cloning vector, which is split into two pieces. **C)** The cloning vector is amplified using primers that will add homologous sequences to the chloroplast genome at its termini. It is also split into two fragments that overlap in the *HIS3* marker to reduce the occurrence of false positives during assembly. Here, the forward and reverse primers positioned at the vector’s termini contain 50 bp of homologous sequence to the integration region in the chloroplast genome. **D)** The chloroplast fragments are amplified using primers that add 20- to 40 bp of homologous sequences to the cloning vector at their respective termini. This will enable the integration of the cloning vector into a specific region in the chloroplast genome.

*Note: This overlapping region will be used to generate primers in step A3; the actual length of overlaps between fragments will range from 150 to 300 bp (Figure 1A and B).*

The design principles we followed for splitting the *P. tricornutum* chloroplast genome into overlapping fragments are described in detail in our original article. Some considerations for the design include:

a. The cloning vector for whole-genome assembly should insert into a non-coding region of the genome; the chloroplast fragment termini should not overlap with each other at this insertion site (Figure 1A–D).

b. It is ideal to split the chloroplast genome into as few fragments as possible to increase assembly efficiency. The exact design approach used will depend on the genome structure and content, as well as the polymerase used for amplification. We were able to reliably amplify fragments as large as 18 kb with Takara PrimeSTAR GXL polymerase.

c. Avoid splitting the genome in repetitive regions, as this can lead to complications during yeast assembly (see Troubleshooting 1).

3. Copy the 400 bp (5′ to 3′ orientation) sequence where the fragments overlap and paste this into Primer3web. In the *Product Size Ranges* section, input 150–300, then click *Pick Primers*. This will generate a forward primer and reverse primer; use the forward primer for the “downstream” fragment, and the reverse primer for the “upstream fragment.” This will enable the amplification of fragments that overlap by 150–300 bp (Figure 1A), which is a suitable size for yeast assembly. Repeat this for every overlap region.

*Note: Yeast can assemble fragments that have overlaps as small as 20 bp [13]; however, we recommend using larger overlaps where possible*.

**Troubleshooting:** If Primer3 cannot identify any suitable primers, try selecting a larger region (e.g., 1,000 bp) with the same *Product Size Ranges* input. Alternatively, the overlap region can be shifted elsewhere; some regions of the chloroplast genome are particularly AT-rich, making it difficult to find optimal primers.

a. As mentioned above, the chloroplast fragments should not overlap at the region where the cloning vector will insert. For our design, we chose a non-coding region between fragments 7 and 8 as the integration site. Here, we designed forward and reverse primers that contained ~30 bp of homology to the chloroplast genome, and 40- to 60 bp of homology to the cloning vector termini (Figure 1D, Table 1). We also amplified the cloning vector using the same premise, such that there was ≥80 bp of homology between the respective fragment termini and cloning vector (Figure 1C, Table 1).

*Note: We originally amplified the chloroplast fragments without the additional homology sequences to the cloning vector, but we recommend adding these to improve assembly efficiency.*

b. We chose the cloning vector pPt0521S_URA (GenBank, accession number: KP745602.1) for our design because it contains BAC/YAC elements and an *oriV* sequence (see General note 9). The BAC/YAC elements enable plasmid replication, stability, and selection in both yeast and *E. coli*.

c. We used primers to split the cloning vector in the yeast *HIS3* open reading frame using the primer optimization technique described in step A3. Splitting the cloning vector in the yeast selective marker can help reduce the number of false-positive transformants following assembly.

**Table 1.** Primers used for adding homology to the termini of the cloning vector and chloroplast fragments. Location refers to the coordinates of the chloroplast fragments relative to the reference genome (GenBank, accession number: EF067920.1; Oudot-Le Secq et al. [12]). Base pairs that are complementary to the chloroplast genome are shown in lowercase, whereas base pairs that are complementary to the cloning vector are shown in uppercase.

Amplicon	Location		Primers (5′ to 3′)
	**From**	**To**	
Chloroplast genome fragment 7	77,856	91,605	Forward: tggaatttagttgggttacgc Reverse: AGGGTTATGCAGCGGAAGATaaaaattcgttaattatttactt aatacgaacatttaatttaatttatcaaaagttaaat
Cloning vector fragment 1	91,605	91,606	Forward: ttttgataaattaaattaaatgttcgtattaagtaaataattaacgaatttttATC TTCCGCTGCATAACCCTGCTTCGG Reverse: TTCAGTGGTGTGATGGTCGT
Cloning vector fragment 2			Forward: CAGTAGCAGAACAGGCCACA Reverse: taaaaatttactgaaaaaaatcaaataaacttagagaaagagtaattcttAA ACCAAAGCGGAGTGACTGCAACTAATGA
Chloroplast genome fragment 8	91,606	103,519	Forward: AATTTAATTTTCATTAGTTGCAGTCACTCCGCT TTGGTTTaagaattactctttctctaagtttatttgatttttttcag Reverse: ttatcaccggcaaaaccttc

**B. Obtaining overlapping fragments from the algal chloroplast genome**

1. Isolate high molecular-weight (HMW) DNA (described in Giguere et al. [9])

a. We believe this DNA extraction method can be used for other algal species; however, we have only tested it with *P. tricornutum*, so we will write it as such.

b. Place a mortar and pestle into a -80 °C freezer hours ahead of attempting DNA isolation to ensure that it is sufficiently chilled.

c. Transfer 5 mL of a dense liquid culture of *P. tricornutum* (i.e., ≥ 6 × 10^6^ cells/mL) into 50 mL of L1 media in a 250 mL glass flask. Repeat this across four flasks and grow until a density of 2–4 × 10^6^ cells/mL is reached (~1 week). Place liquid cultures in a chamber at 18 °C with cool white light (i.e., blue-shifted) set to a photosynthetic photon flux density (PPFD) of around 70 μmol/s/m^2^.

*Note:* P. tricornutum *cell density was estimated by counting cells with a hemocytometer. The culture was diluted 100× with L1 media. Then, 10 μL was pipetted into the counting chamber of a Neubauer-improved hemocytometer.*

d. Transfer the cultures to four 50 mL conical tubes and spin at 3,000 RCF for 10 min at 4 °C. Decant the supernatant, ensuring that the pellet is not disrupted.

**Pause point:** The pellets can be left on ice for up to 2 h or frozen at -80 °C at this stage if needed. Frozen pellets can be kept for up to a month. It is ideal to flash-freeze the pellets if possible.

e. Resuspend each pellet with 1 mL of ice-cold TE buffer, then combine the resuspended cells into one conical tube.

f. Fill the pre-cooled mortar with a small volume of liquid nitrogen and begin adding the resuspended cells to it dropwise using a P1000 pipette. Add liquid nitrogen as needed to ensure the cells remain frozen as small beads.

g. Use the pre-cooled pestle to grind the frozen beads into a fine-grit powder, adding liquid nitrogen as needed to keep the cells frozen.

*Note: The purpose of this step is to break down the exterior diatom cell wall, which is otherwise relatively impervious. Be sure to grind the cells sufficiently.*

h. Transfer the frozen powder into a 15 mL conical tube and add 2 mL of CTAB lysis buffer and 10 μL (i.e., 200 μg) of proteinase K solution (20 mg/mL). Mix slowly using end-over-end inversion, then incubate at 37 °C for 15 min.

i. Gently mix the tube using end-over-end inversion once more, then place into a 37 °C incubator for another 15 min.

*Note: As the cells lyse, the suspension will become a lighter green color.*

j. Pellet the cell resuspension by spinning at 6,000 RCF for 5 min. Transfer the lysate to a new 15 mL conical tube.

k. Add one volume of phenol:chloroform:isoamyl alcohol (25:24:1) and mix gently by end-over-end inversion.

**Caution:** Phenol and chloroform are hazardous volatile chemicals; perform this step and subsequent steps in a ventilated fume hood with the appropriate personal protection equipment (PPE) until otherwise mentioned.

l. Centrifuge the sample at 6,000 RCF for 5 min, then carefully transfer the aqueous phase (i.e., top layer) to a new 15 mL conical tube.

m. Add one volume of chloroform:isoamyl alcohol (24:1) and mix gently by end-over-end inversion.

n. Centrifuge the sample at 6,000 RCF for 5 min, then carefully transfer 450 μL of the aqueous phase to a 1.5 mL tube. Repeat this as many times as necessary to remove as much of the aqueous phase as possible without transferring any of the interphase layer. After this point, the samples should no longer contain any chloroform, so all further steps can be conducted at a lab bench.

o. To the 1.5 mL tube(s), add a tenth volume of sodium acetate solution (3 M, pH 5.2) and two volumes of ice-cold 100% ethanol. Mix gently using end-over-end inversion.

**Pause point:** The sample(s) can be left at -80 °C for 1 h or -20 °C overnight to increase the yield of DNA.

p. Centrifuge the sample(s) at 16,000 RCF for 5 min, then decant the supernatant. Invert decanted tubes on a paper towel and dry until all residual ethanol has evaporated.

**Optional:** Use a chilled (i.e., ≤ 4 °C) centrifuge to increase the yield of DNA.

q. Resuspend the pellet with 100 μL of sddH_2_O and store at -20 °C.

**Optional:** Measure the concentration and purity of the genomic DNA using a fluorometer and/or spectrophotometer.

2. PCR amplification of fragments for whole-genome assembly

a. **For the chloroplast fragments:** Dilute the HMW DNA ~100 times in double-distilled water (ddH_2_O) that has been previously sterilized by autoclaving to ensure no nucleases are active. The concentration of the diluted DNA should be between 0.1 and 1 ng/μL.

b. **For the cloning vector:** Dilute a suitable cloning vector ~100–1,000 times in ddH_2_O so that there is less than 1 ng/μL of template DNA.

*Note: In our original article, we used the pCC1BAC-derived cloning vector pPt0521S_URA for the PCR-based approach (GenBank, accession number: KP745602.1). The plasmid pINTO_7/8 is a SapI-domesticated version of pPt0521S_URA that we generated for the pre-cloned approach outlined in our original article; it can be obtained from Addgene (ID: 206431).*

c. Prepare the PCR master mix (ddH_2_O, buffer, dNTPs, template DNA) according to the Takara PrimeSTAR GXL manual. This particular DNA polymerase kit was chosen as it has proven to be reliable at amplifying fragments as large as 18 kb (see General note 1).

d. Validate whether successful amplification took place by loading 1–2 μL the PCR products on a 1% agarose gel.

e. If amplification was successful, purify the PCR products using the BioBasic EZ-10 Spin Column PCR Products Purification kit, following the manufacturer’s guidelines.

**Figure 2.**
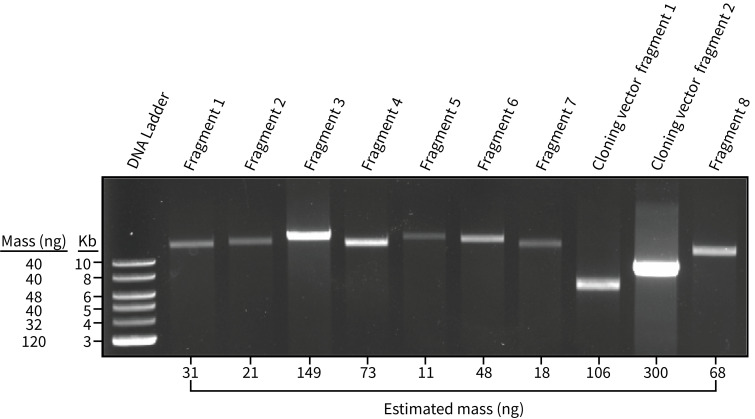
1% agarose gel where 1 μL of the fragments for chloroplast genome assembly (PCR-based approach) were visualized. Fragments 1–8 correspond to amplified regions of the *P. tricornutum* chloroplast genome, whereas the cloning vector fragments correspond to the amplified cloning vector, pPt0521S_URA. The NEB 1 kb ladder plus was used in lane 1 and serves as a reference for estimating the amount of DNA present in the subsequent lanes. Estimated mass values were generated in Image Lab.

3. Preparation of equimolar DNA mixtures for assembly

a. There are many different ways to estimate/measure DNA concentration. The easiest and perhaps most reliable is to use an agarose gel (Figure 2). To do this, load 1 μL of each assembly component into a 1% agarose gel along with 1 μL of an appropriate DNA ladder (e.g., 1 kb plus ladder, NEB).

b. Run the gel until reasonable separation has occurred, then image it using a gel documentation system. Download the .scn file and upload it onto a device that has the Image Lab software installed.

c. Open the .scn file in Image Lab and click on the *Lanes and Bands* button in the *Analysis Tool Box* section. Manually add the correct number of lanes onto the gel image, adjusting the frame as necessary, then manually add the bands for the ladder and sample lanes.

d. Go back to the *Analysis Tool Box* section and click on *Quantity Tools*. At the top of the selection, click *Absolute* and change the units to nanogram, leaving the regression method as linear.

e. Click on the top band of the ladder. The application will ask for a quantity value, which will depend on the specifications of the particular ladder used. For the 1 kb plus ladder, the band at 10 kb corresponds to 42 ng, the band at 6 kb corresponds to 50 ng, and so forth. Add the values for at least three ladder bands that have different masses.

f. Now, when you click on the bands that correspond to your samples, Image Scan will give a predicted value in nanograms. After this window pops up, record the value in a separate document, then press cancel.

*Note: The actual nanograms of DNA will differ from the predicted value. What matters most here is to estimate the amounts of each sample relative to one another. Relative intensity can be gauged by the eye, but using a tool like Image Scan is more accurate.*

g. Input the fragment length and estimated mass into a spreadsheet and perform the series of calculations demonstrated in Table 2. For whole-genome assembly, we created 40 μL assembly mixes. The assembly mix volume can be increased or decreased, so long as there is a sufficient amount (≥ 50 ng) of every assembly fragment; it is better to use more DNA than less. In our original article, we mistakenly reported that we used 50–400 μg of each fragment, when in fact, we used 50–400 ng.

h. Pipette the relative amounts of DNA into a 1.5 mL tube. Store at -20 °C until assembly is performed.

*Note: It is recommended that at least two separate mixtures are prepared in case one of the fragments was not added properly.*

**Table 2.** Calculations to estimate equimolar amounts of DNA from the agarose gel shown in Figure 2. Calculations are demonstrated in the first row containing data (i.e., Fragment 1 row). The relative proportion is calculated by dividing the value of length/mass in a cell by the sum of all values in the length/mass column. Then, the relative proportion value is multiplied by the anticipated final volume for the assembly reaction mixture, which is 40 μL in this example. This generates a proportional amount of DNA to add to the assembly mixture for each respective element. In practice, it is best to add a minimum of 1 μL of each assembly component to the mixture as demonstrated in the adjusted volumes column; this is to avoid pipetting error associated with microvolumes. For assembly of the *P. tricornutum* chloroplast genome, we amplified the genome as eight fragments that were assembled with the cloning vector pPt0521S_URA, which was split into two fragments (cloning vector fragments 1 and 2).

Assembly fragments	Estimated mass (ng)	Length (kb)	Length/mass	Relative proportion	Volume to add to assembly mix (μL)	Adjusted volumes (μL)
Fragment 1	31	14.5	31/14.5 = 0.468	0.468/4.47 = 0.105	40 × 0.105 = 4.2	4.2
Fragment 2	21	15.3	0.729	0.163	6.5	6.5
Fragment 3	149	17.3	0.116	0.026	1.0	1.0
Fragment 4	73	14.6	0.200	0.045	1.8	1.8
Fragment 5	11	17.6	1.600	0.358	14.3	14.3
Fragment 6	48	15.7	0.327	0.073	2.9	2.9
Fragment 7	18	13.8	0.767	0.172	6.9	6.9
Cloning vector, fragment 1	106	6.5	0.061	0.014	0.5	1.0
Cloning vector, fragment 2	300	8	0.027	0.006	0.2	1.0
Fragment 8	68	11.9	0.175	0.039	1.6	1.6
Total	N/A	N/A	4.47	N/A	40.0	41.2

**C. Assembling the whole genome in *S. cerevisiae* (i.e., yeast assembly)**

Adapted from the protocol described in Karas et al. [10]. Perform all steps aseptically.

1. Streak out a glycerol stock of *S. cerevisiae* strain VL6-48 onto 1% (w/v) bacto-agar YPAD plates and incubate at 30 °C until single colonies form (typically two days).

*Note: Once struck out, plates containing VL6-48 can be parafilmed and kept at 4 °C for months. It is good practice to use recently passaged yeast ahead of assembly. The colonies should be white in color; if colonies appear pink, there is likely insufficient adenine hemisulfate present in the YPAD media.*

2. At the beginning of the day, inoculate a single VL6-48 colony into 20 mL of 2× YPAD in a sterile 100 mL flask. Grow at 30 °C with 225 rpm shaking.

*Note: If contamination issues occur, try adding ampicillin (100 μg/mL) to the media.*

3. At the end of the day, measure the optical density at 600 nm wavelength (OD_600_). Dilute the culture into 50 mL of 2× YPAD such that the culture will reach an OD_600_ of 2.5–3.0 at the desired harvest time on the following day. Grow at 30 °C with 225 rpm shaking.

*Note: The doubling time of VL6-48 can vary between 1.5 and 2.5 h. It is good practice to set up at least two additional cultures in case the yeast grows faster or slower than anticipated. Also, it is important to use 2× YPAD for culturing the yeast at this stage as growth in richer media has been shown to increase assembly efficiency.*

a. We measure OD_600_ using 1 mL polystyrene cuvettes in a spectrophotometer. For accurate measurements, the OD_600_ value should fall between 0.2 and 1.0. At this stage, the starter culture is often below an OD_600_ of 1.0, so we use 1 mL of undiluted culture for measuring. If the culture is anticipated to have a density >1.0, we dilute it 2–10× using 2× YPAD (e.g., 10× = 100 μL of culture + 900 μL 2× YPAD).

b. When preparing additional cultures, we recommend setting them up so that one is at least a doubling unit ahead (i.e., more dense) and the other is at least a doubling unit behind (i.e., less dense) than the culture that is predicted to reach the desired OD.

c. Every 50 mL culture can be used to perform 10 assembly reactions. If including a positive and negative control (see General note 3), this means that eight separate constructs can be assembled.

4. When the culture reaches an OD_600_ of 2.5–3.0, transfer it to a 50 mL conical tube and centrifuge the cells at 2,500 RCF for 5 min at 10 °C. Decant the supernatant.

5. Resuspend the pellet in 20 mL of ddH_2_O by vortexing, then add an additional 30 mL of ddH_2_O. Invert to mix, then centrifuge at 2,500 RCF for 5 min at 10 °C. Decant the supernatant.

6. Resuspend the pellet in 20 mL of 1M D-sorbitol by vortexing.

**Pause point:** The resuspended cell pellet can be kept at 4 °C for up to 16 h without impacting assembly efficiency.

7. Add an additional 30 mL of 1M D-sorbitol. Invert to mix, then centrifuge at 2,500 RCF for 5 min at 10 °C. Decant the supernatant.

a. While centrifugation is taking place, prepare at least five cuvettes. In the first cuvette, mix 500 μL of ddH_2_O with 500 μL of 1M D-sorbitol. This will serve as a blank. Into the other cuvettes, add 800 μL of 1M D-sorbitol (**mixture A**) or ddH_2_O (**mixture B**). These cuvettes will be used to estimate the rate of the spheroplasting.

8. Resuspend the pellet in 20 mL of SPEM solution by vortexing. Add 30 μL of 2-mercaptoethanol and mix thoroughly by vortexing once more, then add 40 μL of zymolyase solution. Mix gently by end-over-end inversion, then place at 30 °C shaking at 75 rpm.

**Critical:** Once the zymolyase is added, it is crucial to be very gentle with the yeast. The spheroplasted cells are very sensitive to physical force; do not vortex the cells or shake too vigorously!

**Caution:** β-mercaptoethanol is a hazardous volatile chemical. We recommend performing this step and all proceeding steps in a biosafety cabinet until otherwise mentioned. Ensure that correct PPE is worn.

9. After 10 min has passed, gently transfer 200 μL of spheroplasted cells into the cuvette containing 800 μL of mixture A. Repeat this for mixture B. Then, place a piece of parafilm over the openings of the cuvettes and invert 3–5 times to mix.

10. Measure the OD_600_ and calculate the ratio of mixture A/mixture B. If the ratio is between 1.8 and 2.0, proceed to the next step. If the ratio is lower than this, continue to incubate the cells, checking on the optical density every 5–10 min (or as necessary).

**Critical:** Do not over-spheroplast the cells.

a. While measuring the OD, keep the cells stationary and at room temperature to avoid over-spheroplasting.

b. When first attempting this protocol, we suggest measuring and recording the A/B ratio before placing the culture into the incubator (i.e., time-point zero). These values can serve as a useful reference for gauging the rate of spheroplasting.

c. Spheroplasted cells are more apt to burst when placed in ddH_2_O compared to sorbitol; thus, as the reaction occurs, the ratio of mixture A/ mixture B should increase. If the cells over-spheroplast, this ratio will be greater than 2.0.

d. If an A/B ratio of 1.8–2.0 is not reached after 30 min, add another 15–30 μL of zymolyase solution. Incubate the cells as before and measure the optical density every 5 min.

11. Add 30 mL of 1M D-sorbitol to the cell mixture, then gently invert to mix. Centrifuge at 1,000 RCF for 5 min at 10 °C. Gently decant the supernatant.

*Note: The cell pellet is very fragile and may break; be careful during this step and all subsequent steps.*

**Caution:** Collect the supernatant, which contains 2-mercaptoethanol, in an appropriate vessel for hazardous disposal. The cell pellet should only contain trace amounts of 2-mercaptoethanol, so all further steps can be conducted at a lab bench.

12. Add 10 mL of 1M D-sorbitol, then gently resuspend the pellet by passing it 5–10 times through a 10 mL pipette.

*Note: A P-1000 pipette can be used in lieu of a serological pipette, though the latter is more gentle as it has a wider opening.*

13. Add an additional 30 mL of 1M D-sorbitol and gently invert to mix, then centrifuge as in step C12. Decant the supernatant gently.

a. While centrifugation occurs, prepare aliquot(s) of the PEG-8000 solution and equilibrate to 37 °C (see General note 2). The cell mixture will be resuspended with 1 mL of PEG-8000 solution at a later step.

14. Resuspend the pellet with 2 mL of STC solution, then incubate at room temperature for 10–20 min. As the cells incubate, remove the DNA assembly mix from -20 °C and allow it to thaw at room temperature. Prepare additional 1.5 mL tubes for the positive and negative control (see General note 3).

15. For each assembly reaction, combine 200 μL of the spheroplasted yeast with the respective volume of pre-mixed DNA. Mix by gently flicking the tube, then incubate at room temperature for 5 min.

16. Add 1 mL of 37 °C PEG-8000 solution to each assembly mixture and mix gently by inverting the tube 6–10 times. Incubate at room temperature for 15–20 min.

17. Centrifuge at 1,500 RCF for 7 min at room temperature. Carefully remove the supernatant with a P-1000 pipette.

18. Resuspend the pellet in 800 μL SOS media and incubate at 30 °C for at least 30 min.

*Note: The cells can be left to incubate for up to 1 h without impacting assembly efficiency.*

a. While the assembly mixtures incubate, melt the drop-out media required for assembly; we used -HIS/URA drop-out media for assembling the *P. tricornutum* chloroplast genome, but this will vary depending on which auxotropic selection markers are present in the construct design. Drop-out media can be melted by microwaving on a low-power setting to avoid bubbling over and burning. Ensure that 1M D-sorbitol is present in the media, as the spheroplasted cells will burst if it is absent.

b. Pour 8 mL aliquots of molten media into sterile 15 mL conical tubes, then place in a water bath set to 50 °C. Then, pour 20 mL aliquots of drop-out media into sterile Petri dishes (100 mm diameter). There should be two conical tubes and two plates per assembly reaction, as well as a conical tube and plate for each of the controls.

19. For each assembly reaction, combine 100 μL of the spheroplasted cells/DNA mixture with 8 mL of equilibrated drop-out media, then invert 3–5 times. Pour the mixture onto a Petri dish containing 20 mL of cooled drop-out media. Repeat this using the remaining 700 μL of cells (see General note 4).

a. The assembly plates will consist of a 20 mL bottom layer of drop-out media and an 8 mL top layer of drop-out media mixed with transformed yeast cells. The bottom layer of agar helps to prevent the plate from desiccating during incubation.

b. The spheroplasted yeast cells are very delicate. The agar matrix provides structural support for the cells during the initial stages of cell wall recovery, which is believed to enhance transformation efficiency.

c. Alternatively, the 8 mL of drop-out media mixed with transformed cells can be poured into an empty plate. After 1 day of incubation at 30 °C, 8 mL of liquid drop-out media can be added to the plate, which is then parafilmed to prevent desiccation. Here, colonies will grow into the top liquid, forming a pool of yeast transformants. To obtain single yeast colonies, it will be necessary to plate dilutions of the top liquid on suitable drop-out media plates after sufficient growth has occurred (i.e., > 4 days).

20. Once sufficiently dry (approximately 5 min), place the plates into a bag and move to a 30 °C incubator. For whole-genome assembly, colonies should emerge after 4–5 days. If colonies do not appear, see Troubleshooting 1.

**D. Isolating and screening assembled constructs from *S. cerevisiae***

**Figure 3.**
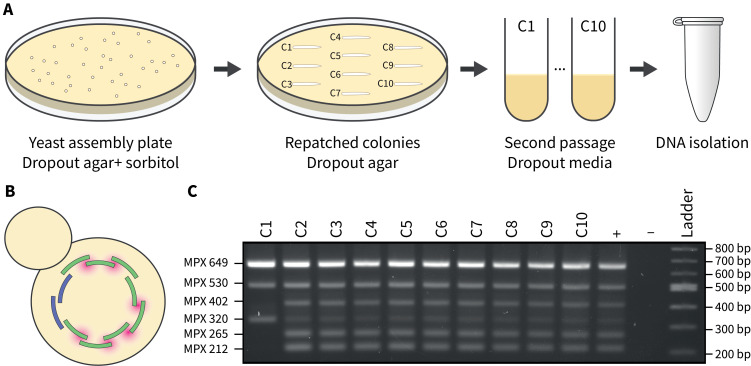
Passaging and screening yeast transformants post-assembly. A) At least ten individual yeast colonies are picked from the assembly plate and struck onto a fresh plate of drop-out media. After sufficient growth (~2 days), colonies are transferred to liquid drop-out media and grown overnight. The next day, DNA isolation via alkaline lysis is performed. **B)** Yeast transformants are screened using a multiplex PCR assay that spans the junctions where chloroplast fragments recombine (highlighted in pink). **C)** A 2% agarose gel where 2 μL of the multiplex PCR reactions were visualized. Transformants that demonstrate the expected multiplex banding pattern (C2–C10) suggest that the whole chloroplast genome has been assembled. The positive control consists of genomic DNA from *P. tricornutum*, and the negative control has no DNA added.

1. Isolating DNA from yeast transformants via alkaline lysis

a. Repatch at least 10 single transformants onto drop-out plates and incubate at 30 °C (Figure 3A, see General notes 5 and 6).

*Note: At this point, the yeast cells will have recovered their cell walls; use drop-out agar that does not have D-sorbitol added, as it will interfere with cell growth if present (see Recipes).*

When repatching, pick a single colony using a sterile pipette tip and make a 1–2 cm patch on the agar plate. Repeat this step until all 10 colonies have been patched on the plate. It is not necessary to streak out the transformants to obtain single colonies.

b. Once sufficient growth has occurred (approximately 2 days), inoculate each transformant into 5 mL of liquid drop-out media in 15 mL conical tubes. Place at 30 °C with shaking at 225 rpm overnight.

When inoculating, touch a sterile pipette tip to the end of the patch from step D1a and drop it into the liquid media.

c. The next day, pellet the cells by centrifuging at 3,000 RCF for 5 min at room temperature. Decant the supernatant.

d. Resuspend the pellet with 240 μL of P1 buffer, 5 μL of 2-mercaptoethanol, and 5 μL of zymolyase solution. Thoroughly mix and then transfer to a 1.5 mL tube.

**Caution:** 2-mercaptoethanol is a hazardous volatile chemical. We recommend performing this step and all future steps in a fume hood until otherwise mentioned. Ensure that correct PPE is worn.

e. Add 250 μL of P2 buffer and gently invert 6–10 times to mix. Incubate for 2 min at room temperature.

f. Add 250 μL of P3 buffer and thoroughly mix by inversion. Centrifuge at the maximum speed (e.g., 16,000 RCF) for 10 min at room temperature.

g. Transfer the supernatant (approximately 750 μL) to a clean 1.5 mL tube, then add 750 μL of -20 °C 100% isopropanol. Invert to mix, then centrifuge as in step D1f.

**Pause point:** The sample(s) can be left at -80 °C for 1 h or -20 °C overnight to increase the yield of DNA. Using a chilled centrifuge can also increase DNA yield.

h. Decant the supernatant, then add 500 μL of -20 °C 70% ethanol. Invert to mix, then centrifuge at maximum speed for 5 min at room temperature.

**Optional:** Use a chilled centrifuge to increase DNA yield.

i. Decant the supernatant and allow the DNA pellet to dry thoroughly. At this point, all traces of 2-mercaptoethanol should be gone.

j. Resuspend the pellet with 30–50 μL of ddH_2_O, then store at -20 °C.

**Optional:** Measure the concentration and purity of the DNA using a fluorometer and/or spectrophotometer. Alkaline lysis should yield a final concentration greater than 100 ng/μL.

2. Screening DNA for the assembly of complete chloroplast genomes

a. Prepare the QIAGEN multiplex (MPX) master mix according to the manufacturer’s guidelines (Figure 3B, see General note 7).

*Note: The primer pairs used to screen for assembly of the whole genome span 6 of the 8 overlapping junctions between the chloroplast fragments. The design of these primers is described in detail in our original manuscript.*

b. Use 1 μL of 10× diluted yeast DNA as the template for the reactions. For a positive control, use 1 μL of 100× diluted genomic DNA from *P. tricornutum*. For the negative control, use 1 μL of ddH_2_O.

*Note: Including both controls is necessary. Any amplification of the negative control suggests contamination or carry-over of DNA during pipetting, see Troubleshooting 2.*

c. Perform 30 cycles of the MPX reaction. Increase to 35 cycles if necessary.

*Note: Increasing the number of cycles heightens the risk of false positives.*

d. Load 2 μL of amplified MPX DNA on a 2% agarose gel with an appropriate DNA ladder (Figure 3C).

*Note: The expected sizes for the amplicons fall within 100–700 bp, so it is necessary to use a 2% agarose gel for proper separation. If amplification fails for the yeast transformant DNA, see Troubleshooting 3.*

3. Alternative screening method for assembly of the complete chloroplast genomes

a. If it is too cumbersome to perform alkaline lysis for several yeast transformants, an alternative method is to perform a rapid heat lysis of the cells.

b. Follow steps D1 (a and b) to passage yeast transformants. Instead of transferring the cells to liquid drop-out media, passage onto a plate for a second time.

c. Once sufficient growth has occurred (approximately 2 days), touch a pipette tip to the passaged yeast colony, then place the pipette tip into 10 μL of TE buffer in a microcentrifuge tube. Let it sit for 2–5 min before disposing of the pipette tip.

d. Vortex the tubes to sufficiently mix the contents, then incubate at 95 °C for 10 min.

*Note: This heat treatment will lyse the cells, causing the release of DNA into the TE buffer.*

e. Centrifuge the tubes until the cell debris has pelleted, then use 1 μL of the supernatant as template in the MPX reaction (step D2b).

*Note: We only recommend using this method as a preliminary screen for successful transformants. The template DNA will not be of high quality and may lead to false negatives.*

**E. Transforming DNA from *S. cerevisiae* to *E. coli* via electroporation**

*Note: Perform all steps aseptically.*

**Figure 4.**
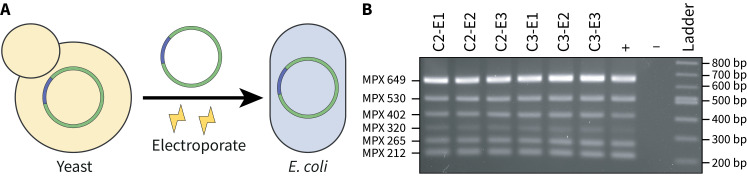
Screening *E. coli* transformants post-transformation with yeast-assembled DNA. A) DNA isolated from yeast transformants that screen positively is electroporated into *E. coli* for further analysis. **B)** A 2% agarose gel where 2 μL of the multiplex PCR reactions were visualized. All *E. coli* transformants demonstrate the expected multiplex banding pattern, suggesting that the whole chloroplast genome has been successfully transformed. The positive control consists of total DNA isolated from *P. tricornutum* and the negative control has no DNA added.

1. Use DNA from yeast transformants that screened positively for the presence of the whole chloroplast genome (Figure 4A).

2. Place sterile 2 mm electrocuvettes on ice.

*Note: It is important to keep everything cold, as this prevents DNases from degrading the DNA during transformation.*

3. Thaw a tube of EPI300 electrocompetent cells (Lucigen) on ice for approximately 10 min (see General note 8).

4. Once thawed, mix 25–50 μL of cells with 1–2 μL of isolated yeast DNA. Flick to mix, then incubate on ice for 5 min.

*Note: It is ideal to use 50 μL of cells; however, 25 μL will suffice if needed. Use 2 μL of yeast DNA if the concentration appears low.*

a. It is good practice to include positive and negative controls when conducting electroporation. Use 1 μL of plasmid DNA that contains the same marker(s) as the assembled construct for the positive control. The negative control will consist of cells with no DNA added.

5. Transfer the mixture to a chilled 2 mm electrocuvette, ensuring that the mixture sits evenly across the bottom of the cuvette and that there are no air bubbles present.

6. Pulse in an electroporator set to 2.5 kV with a capacitance of 25 μF and resistance of 200 Ω.

7. Add 1 mL of SOC media to the cuvette, then pipette up and down 3–5 times to resuspend the cells as they will be stuck to the bottom of the electrocuvette. Transfer as much as possible to a 1.5 mL tube, then place the mixture at 37 °C with shaking at 225 rpm for 1 h.

*Note: The electrocuvettes can be washed and reused several times if properly cared for.*

a. It is challenging to remove the whole reaction volume from the cuvette after resuspension. We find that 800–900 μL of resuspended cells are typically transferred to the 1.5 mL tube.

8. Individually plate 100 and 700 μL of the cells across two separate 1.5% agar (w/v) LB plates supplemented with chloramphenicol (15 μg/mL). Once the plates are dried, transfer to 37 °C. Colonies should emerge within 24 h (see General note 4).

**F. Isolating and screening assembled constructs from *E. coli***

1. Isolating DNA from *E. coli* transformants via alkaline lysis

a. Inoculate a single *E. coli* colony into 5 mL of LB supplemented with chloramphenicol (15 μg/mL). Grow overnight at 37 °C shaking at 225 rpm.

*Note: Do this for at least three* E. coli *colonies per transformation.*

b. The next day, inoculate 500 μL of saturated culture into 5 mL of LB supplemented with chloramphenicol (15 μg/mL) and L-arabinose (100 μg/mL). Grow for 5 h at 37 °C on a shaker set to 225 rpm, ensuring that the cultures are well aerated (see General note 9).

c. Centrifuge 1–5 mL of the culture (1.5 mL is usually enough) at maximum speed for 2 min. Decant the supernatant.

d. Resuspend the pellet with 250 μL of P1 buffer, ensuring that the cells are mixed thoroughly.

e. Add 250 μL of P2 buffer, then invert 6–10 times to mix. Incubate at room temperature for 10 min.

f. Add 250 μL of P3 buffer and thoroughly mix by inversion. Centrifuge at the maximum speed (e.g., 16,000 RCF) for 10 min at room temperature.

g. Transfer the supernatant (approximately 750 μL) to a clean 1.5 mL tube, then add 750 μL of -20 °C 100% isopropanol. Invert to mix, then centrifuge as in step F1f.

**Pause point:** The sample(s) can be left at -80 °C for 1 h or -20 °C overnight to increase the yield of DNA. Using a chilled centrifuge can also increase DNA yield.

h. Decant the supernatant, then add 500 μL of -20 °C 70% ethanol. Invert to mix, then centrifuge at maximum speed for 5 min at room temperature.

**Optional:** Use a chilled centrifuge to increase DNA yield.

i. Decant the supernatant and allow the DNA pellet to dry thoroughly.

j. Resuspend the pellet with 30–50 μL of ddH_2_O, then store at -20 °C.

**Optional:** Measure the concentration and purity of the DNA using a fluorometer and/or spectrophotometer.

2. Screening DNA for the assembly of complete chloroplast genomes

Follow the same approach for screening yeast transformants (step D2) but dilute the template DNA 100–1,000× as it will be more abundant (Figure 4B).

3. Alternative screening method for assembly of complete chloroplast genomes

Follow the same approach for screening yeast transformants (step D3).

4. Isolating *E. coli* DNA for sequencing

Whole-plasmid sequencing is the gold standard for confirming that an assembly is correct. The DNA prepared through the alkaline lysis method works sufficiently for initial screens but is often too poor quality for sequencing due to the carryover of salts, RNA, and shorn genomic DNA during isolation. We used the QIAGEN large construct kit to purify the constructs from *E. coli* transformants that diagnostically appeared to have the correctly assembled genome.

## Data analysis

The majority of data processing and analysis in our original article occurred post–yeast assembly. Here, we performed sequence alignments to investigate if any major rearrangements or mutations accrued when cloning the genome. We also assessed the burden and long-term stability of the chloroplast genome when maintained in *E. coli* under low-copy and high-copy number replication. For the methods presented in this article, there is very little data analysis to be conducted, as the focus is on performing a technically challenging method. Once the genome is assembled and diagnostically validated, we recommend performing next-generation sequencing of a few representative yeast and/or *E. coli* transformants to confirm that the entirety of the construct has been successfully cloned.

## Validation of protocol

We repeated the assembly of the *P. tricornutum* chloroplast genome several times in our original article (Table 3). Though there were varying degrees of efficiency, in every iteration, there were at least 20 yeast colonies to screen post-assembly. We MPX-screened 10 yeast colonies per assembly method, with 90%–100% of the transformants demonstrating the expected banding pattern for assembly of the whole chloroplast genome. For each assembly method, DNA from two prospective yeast colonies was electroporated into *E. coli*. Three colonies were screened per transformation, with 100% of the transformants demonstrating the expected MPX banding pattern. We conducted whole-plasmid sequencing for at least one *E. coli* colony per assembly, all of which demonstrated the cloned chloroplast genome in its entirety. In the time since, we have used this method to assemble several permutations of the chloroplast genome, including halved-, quartered-, and two-third versions.

**Table 3.** Number of colony-forming units following assembly of the whole chloroplast genome in yeast. Derived from supplementary table S5 of Walker et al. [14].

Assembly approach	Volume of spheroplasts plated	
	700 μL	100 μL
PCR-based approach	361	38
Pre-cloned approach, individual digestion of plasmids	47	2
Pre-cloned approach, one-pot digestion of plasmids	15	5
Combinatory approach for assembling pPt_Sap	20	2

## General notes and troubleshooting

**General notes**

1. We followed these principles when amplifying fragments of the chloroplast genome using the Takara PrimeSTAR GXL kit:

a. When first testing primers, it is suitable to perform 10–25 μL reactions to conserve reagents.

b. When PCR-amplifying fragments greater than 5 kb, we use the fast protocol, which requires 2 μL of enzyme per 50 μL reaction. This amplifies DNA at a speed of 1 kb per 10 s.

c. If the PCR primers have been optimized using software like Primer3, we find that an annealing temperature of 60 °C works reliably. For primers that are not optimized, it may be necessary to vary the annealing temperature from as low as 50 °C, if no amplification occurs, and up to 68 °C if off-target bands are present.

d. For all of the PCR-amplified fragments, we began with 25 cycles and increased up to 35 cycles if amplification was insufficient. If you want to conserve reagents, you can place the reaction tube(s) back into the thermocycler for an additional 5–10 cycles. This only works if the tube(s) have not been frozen or left at room temperature for several hours.

2. PEG-8000 is a critical reagent in yeast assembly; it is important to purchase high-quality PEG from a reliable distributor. When testing out this protocol for the first time, we recommend purchasing PEG from more than one vendor to see which works best in your hands. As an anecdotal example, we observed nearly 10 times more yeast transformants when using the Fisher Scientific PEG (catalog number: BP233-1) compared to the Bio Basic PEG (catalog number: PB0433).

a. Make sure to store the PEG solution at 4 °C and test the pH of the solution ahead of use to ensure that it remained around a pH of 8.0 during storage.

b. When performing assembly, we recommend aseptically transferring an aliquot of PEG solution into a 15 mL conical tube that can then be equilibrated to 37 °C and disposed of after use. This is to avoid repetitively exposing the PEG solution to 37 °C as this can cause depolymerization over time. We also recommend keeping the PEG solution in an air-tight bottle as air exposure can lead to oxidation and depolymerization.

3. It is good practice to include positive and negative controls when performing assembly. Here, the negative control consists of spheroplasted yeast with no DNA added. The positive control consists of spheroplasted yeast mixed with at least 100 ng of a suitable control plasmid. This plasmid should contain the same selection marker(s) as the construct that you are assembling, as well as all other necessary elements for plasmid maintenance and replication in yeast and *E. coli*.

4. It can be beneficial to plate both 100 and 700 μL volumes of the cell mixtures. If assembly or transformation is very efficient, it will be easier for colonies to emerge when 100 μL of the reaction volume is plated as there will be less crowding. Conversely, if assembly or transformation is not efficient, then it is advantageous to have 700 μL of the reaction volume plated as this will give rise to more colonies. For the positive and negative controls, it is fine to plate the whole reaction volume (800 μL).

5. The cloned *P. tricornutum* chloroplast genome is ~130 kb, which is too large for most commercial spin-kit columns. We were able to successfully isolate the cloned genome from yeast and *E. coli* using alkaline lysis; however, this may not be possible for chloroplast genomes larger than this. For these cases, it may be necessary to use a specialized kit designed for the isolation of large plasmids.

6. Make sure to passage *S. cerevisiae* or *E. coli* transformants at least twice before conducting the multiplex screen. Any residual DNA left over from assembly or transformation can be carried over and, when screened, lead to false positive results. We recommend transferring as few cells as possible when passaging to avoid carry-over of this environmental DNA.

7. In a multiplex PCR reaction mixture, some primer pairs will amplify better than others. It may be necessary to increase or decrease the relative amounts of the primer pairs so that all of the amplicons have similar levels of amplification. This can be estimated by looking at the relative intensities of the amplicons after visualizing them on a 2% agarose gel.

a. An economical alternative to the QIAGEN MPX kit is the Takara SuperPlex kit. Though we did not use this in our original article, we have used the SuperPlex kit in the time since, and it appears to be as accurate and reliable as the QIAGEN kit.

b. It is important to include high-quality genomic DNA as a positive control for all multiplex reactions. It is also critical to include a negative control; here, we used 1 μL of TE buffer.

8. We performed transformation of the whole chloroplast genome using homemade electrocompetent EPI300 cells. Homemade cells are considerably less efficient than their commercial counterparts; with that being said, we were routinely able to get between 50 and 200 *E. coli* transformants, which was sufficient for our screening purposes. We mention this as commercial cells are notoriously expensive. We use the Warren [15] protocol to prepare large batches of electrocompetent EPI300 cells, which are stored in 50 μL aliquots at -80 °C until use.

9. We chose the EPI300 *E. coli* strain because it is highly competent and contains an arabinose-inducible mutant *trfA* gene integrated into its genome. When induced, *trfA* will lead to high-copy number expression of plasmids carrying an *oriV* (e.g., pCC1BAC-derived plasmids). This can be useful when trying to isolate large quantities of the construct from *E. coli*; however, high-copy level expression interferes with cell growth and can cause plasmid instability if sustained over several generations. Without supplemental arabinose, pCC1BAC plasmids are stably maintained as a single copy in EPI300. It is advisable to only induce an EPI300 culture for a few hours before harvesting the cells for DNA isolation. If your cloning vector does not contain an *oriV*, there is no need to supplement LB with L-arabinose.

**Troubleshooting**

1. Chloroplast genomes are not built the same across all photosynthetic eukaryotes; they can vary widely in size, gene content, repetitive regions, and more. If yeast assembly fails, here are a few things to consider:

a. **Was there a positive control during assembly?** The positive control provides a mechanism for ensuring that assembly worked; without this, it is hard to say if assembly failed due to the inability to correctly recombine the DNA fragments, over-spheroplasting, missing media elements (e.g., D-sorbitol), so on and so forth. When first attempting this method, we recommend preparing a large stock of a suitable plasmid that can be used as a positive control in every assembly. You should expect to see several hundreds to thousands of transformants on the positive control plate if the assembly was efficient. It is ideal to use a positive control plasmid that has the same yeast selection marker(s) as the construct you are trying to assemble.

b. **What is the G+C content of the genome?** It is known that DNA with low G+C content (≤30%) is challenging to clone and maintain in *E. coli*, whereas large fragments (≥100 kb) with high G+C content (≥40%) are challenging to clone in yeast [16]. If the G+C content is greater than 40%, it may be necessary to assemble portions of the genome at a time or add additional yeast replication origins throughout the genome [17]. Conversely, if the G+C content is lower than 30%, it may not be possible to transform and stably maintain the genome in *E. coli*.

c. **Are there any toxic genes in the genome?** Toxic genes encode proteins that serve a role in the host organism (or organelle, in this case) but cause disruption when expressed in other systems [18]. Also, regions with high A+T content can contain spurious open reading frames, which can be unexpectedly toxic when cloning [19]. This can be resolved by splitting the genome into multiple fragments and individually assembling each fragment with a suitable cloning vector in yeast [20]. The fragment containing the toxic gene should not give rise to many, if any, yeast and/or *E. coli* transformants compared to the other regions of the genome. The problematic fragment can be split further until the exact location of the toxic gene is identified, allowing for its targeted removal.

d. **How big is the genome?** The *P. tricornutum* chloroplast genome is small (117 kb) when compared to most plant and algal species. For genomes larger than this, it may be necessary to introduce additional origins of replication and other plasmid maintenance elements (e.g., markers) throughout the genome to ensure that it can be stably replicated and maintained in yeast and *E. coli*. This issue was encountered and overcome by O’Neill et al. [8] when cloning the 204 kb chloroplast genome of *C. reinhardtii*. A past study has shown that a 500 kb algal chromosome can be cloned in yeast and moved to *E. coli*, which is much larger than the vast majority of chloroplast genomes, so we believe this should not pose a major impasse [21].

e. **How repetitive is the genome?** The *P. tricornutum* chloroplast genome has two large inverted repeat (IR) regions that we had to account for when designing our assembly strategy, as these regions can easily recombine in yeast. Other chloroplast genomes may have larger IR regions or may contain multiple dispersive repetitive regions. It is important to take this into consideration when designing where the fragment termini are positioned and where the markers for yeast and *E. coli* will be integrated.

2. The QIAGEN MPX kit is very sensitive and can amplify trace amounts of DNA that are aerosolized during pipetting. If amplification occurs in the negative control, we suggest repeating the MPX reaction using a different set of pipettes. We also recommend using the best pipetting practices to prevent the aerosolization and carry-over of DNA; if possible, use barrier pipette tips (e.g., VWR, catalog number: 89082-364).

3. Alcohols, salts, and cell debris carried over during DNA isolation via alkaline lysis can interfere with the MPX DNA polymerase. If no amplification occurs when screening the yeast or *E. coli* transformants, try diluting the DNA another ten-fold and see if that restores amplification. If it does not resolve the issue, we recommend checking the DNA concentration and purity using a spectrophotometer. If the concentration and/or purity ratios are poor, try re-isolating the DNA and reperform the MPX reaction.
